# Risks of all-cause death and completed suicide in patients with schizophrenia/schizoaffective disorder treated with long-acting injectable or oral antipsychotics: A population-based retrospective cohort study in Taiwan

**DOI:** 10.1192/j.eurpsy.2021.2258

**Published:** 2021-12-13

**Authors:** Chao-Hsiun Tang, Shih-Pei Shen, Min-Wei Huang, Hong Qiu, Sayuri Watanabe, Choo Hua Goh, Yanfang Liu

**Affiliations:** 1 School of Health Care Administration, College of Management, Taipei Medical University, No. 172-1, Section 2, Keelung Road, Taipei 106, Taiwan; 2 Chiayi Branch, Taichung Veterans General Hospital, No. 600, Section 2, Shixian Road, West District, Chiayi City 60090, Taiwan; 3 Global Epidemiology, Janssen Research & Development, 1125 Trenton-Harbourton Road, Titusville, New Jersey 08560, USA; 4 Research & Development, Janssen Pharmaceutical K.K., 5-2 Nishi-kanda 3-Chome, Chiyoda-ku, Tokyo 101-0064, Japan; 5 Global Epidemiology, Janssen Research & Development, 2 Science Park Drive, Singapore 118222, Singapore

**Keywords:** Cohort study, incidence, mortality, paliperidone, risk

## Abstract

**Background:**

Long-acting injectable (LAI) antipsychotics improve medication adherence in patients with schizophrenia and extend the duration of therapeutic drug levels but with administration of an increased dose. Real-world mortality data in patients prescribed LAIs are lacking. We conducted a population-based cohort study to estimate and compare the incidence rates of all-cause death and completed suicide in patients with schizophrenia/schizoaffective disorder exposed to LAIs and oral antipsychotics.

**Methods:**

Patients with a diagnosis of schizophrenia/schizoaffective disorder between January 1, 2015 and November 30, 2019 were enrolled from the Taiwan National Health Insurance Research Database and linked to Death Registry records. Eligible patients were new antipsychotic users. Relative risks of death for each antipsychotic compared with oral paliperidone were evaluated using a Cox proportional hazard model adjusted for age, sex, Charlson Comorbidity Index, index year, bipolar or major depressive or other mood disorders, mental disorders due to drug use, and baseline hospitalization frequency.

**Results:**

There were 228,791.08 person-years of follow-up (mean 2.48 years). The incidence rates of all-cause death in users of LAI paliperidone administered monthly (PP1M) and every 3 months (PP3M) were 7.40/1,000 person-years (95% confidence interval 5.94–9.11) and 9.93 (5.88–15.79), respectively. The incidences of completed suicide were 2.03/1,000 person-years (1.32–2.99) and 3.10 (1.14–6.88), respectively. No significant associations were observed between PP1M and PP3M compared to oral paliperidone in incidences of all-cause death or for completed suicide.

**Discussion:**

No increased risk of all-cause death or completed suicide was observed in users of antipsychotic LAIs, including PP1M and PP3M.

## Introduction

Schizophrenia affects 0.28% of the global population and has a prevalence in Asia of 0.42% [1]. The life span of individuals with schizophrenia can be up to 20 years shorter than the general population, and the risk of death is raised by two- to threefold [2–4]. Lifestyle factors such as poor dietary habits, substance abuse, low levels of exercise, an increased incidence of comorbidities including cardiovascular disease, noncompliance with treatments, and an elevated risk of mortality from suicide are all likely contributors [5–7].

Long-term antipsychotic treatment is recommended for all persons with schizophrenia, and poor treatment adherence is one of the strongest risk factors for relapse [8,9]. Maintaining long-term adherence to treatment is one of the most challenging aspects in the management of schizophrenia [10,11]. Treatment gaps as short as 1–10 days are associated with a twofold increase in hospitalization risk [12]. In this setting, long-acting injectable (LAI) antipsychotics can improve medication adherence by lengthening the dose interval and providing an extended duration of therapeutic drug levels. Currently available LAIs include typical and atypical antipsychotics with administration intervals that vary from 4 to 8 weeks, and 3 months for LAI paliperidone (PP3M). Real-world data show that LAIs improve treatment adherence, prevent relapse, reduce hospitalizations, and lower associated medical costs compared with oral atypical antipsychotics [13,14].

In view of the increased risk of death in persons with schizophrenia, it is important to ensure that new therapies do not contribute to this risk. A meta-analysis of 52 randomized clinical trials found no significant differences in all-cause death or in death due to suicide in users of LAIs or oral antipsychotic drugs [15]. However, comparisons between individual LAIs were not possible due to insufficient data. Real-world data comparing mortality in patients prescribed different LAIs including paliperidone administered monthly (PP1M), which is one of the most frequently used LAIs, and the most recently approved PP3M, are not available.

We conducted a retrospective, population-based cohort study to evaluate and compare the incidence of all-cause death and completed suicide in patients with schizophrenia or schizoaffective disorder who were users of LAI versus oral antipsychotics. In addition, we evaluated the risk of death or suicide for individual LAIs compared to oral paliperidone.

## Methods

### Data source

Taiwan’s single-payer National Health Insurance (NHI) program covers more than 99% of the Taiwan population of 23 million. The National Health Insurance Research Database (NHIRD) includes dates and types of the claims of reimbursed healthcare goods and services, such as procedures, dispensed prescription drugs, and medical services provided to inpatients and outpatients with diagnoses that were coded by the International Classification of Diseases (ICD), Ninth Revision, Clinical Modification (ICD-9-CM) until 2016, and by the 10th version (ICD-10) thereafter. NHIRD-specific drug codes include the drug, route of administration, and strength. A unique and anonymous identifier for each patient links the NHIRD with the Death Registry. Each death certificate in Taiwan is legally required to be registered using ICD-10 codes within 4 weeks after death. All death certificates are reviewed centrally by trained medical registrars. As a result, cause of death coding in Taiwan is considered highly accurate and complete [16].

### Study design and population

This retrospective cohort study enrolled patients ≥18 years of age with a confirmed diagnosis of schizophrenia or schizoaffective disorder (ICD-9: 295.x except 295.7; ICD-10: F20.x) or schizoaffective disorder (ICD-9: 295.7; ICD-10: F25.x), who had at least one record for a prescription of antipsychotics between January 1, 2015 and November 30, 2019. Baseline patient characteristics were recorded over a 12-month period before the index date (the date of the first qualifying prescription of the index antipsychotic). A new user cohort of patients exposed to each antipsychotic was defined as patients who had not received a prescription for the index antipsychotic in the preceding 12 months. All new antipsychotic users were followed up until discontinuation of treatment, switching, death, or end of study.

### Exposure

PP3M was approved for use in Taiwan relatively recently, and patients must have received PP1M for at least four consecutive months before switching to PP3M. Therefore, we defined treatment groups using a hierarchical approach based on treatment guidelines in place during the study period. We enrolled patients in the cohort analysis in the order of those who initiated treatment with atypical LAI, typical LAI, oral atypical, and typical antipsychotics, such that patients were first checked for exposure to PP3M. If “yes,” the patient was enrolled as a new user of PP3M; if “no,” then exposure of that patient to the next drug in the hierarchy was checked, and so on. A schematic view of the hierarchical approach is provided in the Supplementary Material. This approach allowed us to capture new users of antipsychotics with respect to treatment sequence according to local treatment guidelines and the timeline of approval for reimbursement.

### Outcomes

The study outcomes were all-cause death defined as deaths in the Death Registry due to any cause after the index date, and completed suicide (X60–X84, Y87.0; Table S1), in eligible patients who died after the index date. The date of death was extracted from the Registry.

### Statistical analysis

Age and gender, comorbidities, the number of outpatient visits, psychiatric comorbidities, and episodes due to schizophrenia/schizoaffective disorder were summarized during the baseline period. Comorbidities were assessed using the Charlson Comorbidity Index (CCI). Substance abuse increases the risk of suicide and morality in patients with schizophrenia [17,18], but the NHIRD claims database is unable to capture illicit drug use. Despite a low rate of illicit drug use on Taiwan compared with other countries [19], we used ICD codes for mental disorders due to psychoactive substance use (ICD-9: 292, 304, and 305; ICD-10: F11–F19) as a proxy for substance abuse.

Two analytic scenarios were applied to estimate the risk of all-cause death: In the “as-treated” scenario analysis, the occurrence of all-cause deaths was captured during the active treatment follow-up period defined from the index date to the date of the end of drug supply following the last prescription; date of switching to another antipsychotic; date of death; date of disenrollment from the NHIRD; or December 31, 2019, whichever occurred first. The incidence was calculated by dividing the total number of all-cause deaths during the active treatment period by the total follow-up period. A Cox proportional hazard model was used to estimate hazard ratios (HRs) of all-cause deaths among patients who received PP3M, and other individual LAIs in comparison with patients treated with oral paliperidone. Index year, as well as risk factors, such as age, gender, CCI, and comorbid bipolar disorder, major depressive disorder, or other affective mood disorder, and mental disorder due to drug use were adjusted in the analysis.

In the “fixed cohort” scenario analysis, all deaths were counted, even if initial treatment was discontinued or the patient switched to another treatment. Cumulative incidences of all-cause death and completed suicide were calculated by dividing the number of all-cause deaths within 30, 90, and 180 days of the index date by the total number of antipsychotic new users.

A sensitivity analysis was performed in patients with schizophrenia/schizoaffective disorder registered in the Registry of Catastrophic Illness (RCI). Legislated under the NHI, patients with specified major diseases, including schizophrenia/schizoaffective disorders, can apply for a catastrophic illness certificate that exempts them from all co-payments for inpatient and outpatient care. Approval requires strict evaluation and diagnostic validation. All analyses were performed using SAS Version 9.4 (Cary, NC, USA).

## Results

### Patient population

**Figure 1.**
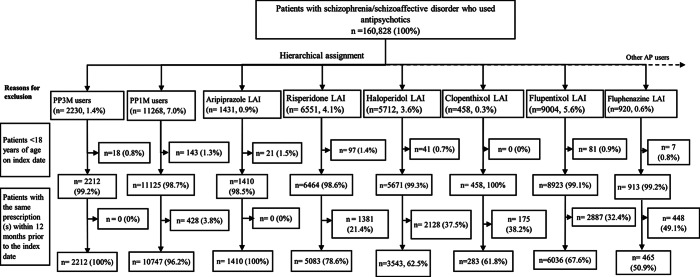
Patient enrollment diagram in users of long-acting antipsychotics (table continued in the Supplementary Material for oral antipsychotics).

There were 160,828 patients with schizophrenia/schizoaffective disorder who met the enrolment criteria and were prescribed an antipsychotic during the period January 1, 2015 to November 30, 2019. After applying exclusion criteria, there were 29,779 new users of LAIs, including PP1M (*n* = 10,747, 36.1% of LAIs), PP3M, (*n* = 2,212, 7.4%), flupentixol (*n* = 6,036, 20.3%), risperidone (*n* = 5,083, 17.1%), haloperidol (*n* = 3,543, 11.9%), and aripiprazole (*n* = 1,410, 4.7%; Figure 1).

The mean age of patients was 43.5 years in PP1M recipients, 44 years in PP3M recipients, and ranged from 41.4 to 45.1 years in users of other LAIs. The mean age of patients using oral antipsychotics ranged from 42.4 (aripiprazole) to 54.5 years (quetiapine; Table 1). The age distribution of patients was younger in users of LAIs compared with oral antipsychotics and included few patients aged above 60 years (Table S2). Proportionally more adults aged 60 years and above were treated with oral quetiapine, whereas proportionally more patients below 30 years of age received oral paliperidone, oral lurasidone, or oral aripiprazole. The mean CCI score ranged from 0.33 to 0.45 in patients prescribed LAIs. The highest mean CCI scores were observed in patients prescribed oral haloperidol (0.83), oral quetiapine (0.93), and oral chlorpromazine (0.93). Psychiatric inpatient episodes during the 12-month baseline period were more frequent in users of LAIs than in users of oral antipsychotics. The percentage of patients using LAIs who had no inpatient episodes within 12 months before the index was lowest in users of clopenthixol (39.2%) and risperidone (40.1%), and highest in users of PP3M (66.1%). In users of oral antipsychotics, 72.6% (paliperidone) to 98.3% (sulpiride) had no inpatient episodes within 12 months before the index date.

**Table 1.** Demographic characteristics of the patient cohort by treatment assignment.^a^

	Long-acting injectable antipsychotic								Oral antipsychotic			
	PP1M (*n* = 10,747)	PP3M (*n* = 2,212)	Aripiprazole (*n* = 1,410)	Risperidone (*n* = 5,083)	Haloperidol (*n* = 3,543)	Clopenthixol (*n* = 283)	Flupentixol (*n* = 6,036)	Fluphenazine (*n* = 465)	Paliperidone (*n* = 6,536)	Risperidone (*n* = 23,113)	Haloperidol (*n* = 12,458)	
Gender, *n* (%)												
Male	5,441 (50.6)	1,145 (51.8)	605 (42.9)	2,699 (53.1)	2,052 (57.9)	181 (64.0)	3,295 (54.6)	278 (59.8)	3,227 (49.4)	11,574 (50.1)	6,649 (53.4)	
Female	5,306 (49.4)	1,067 (48.2)	805 (57.1)	2,384 (46.9)	1,491 (42.1)	102 (36.0)	2,741 (45.4)	187 (40.2)	3,309 (50.6)	11,539 (49.9)	5,809 (46.6)	
Age, years												
Mean ± SD	43.51 (12.24)	43.99 (11.59)	41.44 (12.72)	44.75 (12.74)	45.12 (11.09)	45.04 (9.83)	45.09 (11.87)	44.38 (12.03)	42.57 (13.83)	46.8 (16.29)	49 (15.05)	
Index year, *n* (%)												
2015	1,537 (14.3)	0 (0.0)	0 (0.0)	1,234 (24.3)	891 (25.1)	150 (53.0)	1,496 (24.8)	118 (25.4)	1,646 (25.2)	5,659 (24.5)	3,333 (26.8)	
2016	1,846 (17.2)	0 (0.0)	0 (0.0)	1,091 (21.5)	774 (21.8)	99 (35.0)	1,235 (20.5)	103 (22.2)	1,472 (22.5)	5,370 (23.2)	2,968 (23.8)	
2017	2,406 (22.4)	50 (2.3)	0 (0.0)	994 (19.6)	667 (18.8)	28 (9.9)	1,150 (19.1)	88 (18.9)	1,157 (17.7)	4,595 (19.9)	2,378 (19.1)	
2018	2,664 (24.8)	1,142 (51.6)	359 (25.5)	965 (19.0)	656 (18.5)	6 (2.1)	1,082 (17.9)	88 (18.9)	1,134 (17.4)	4,231 (18.3)	2,043 (16.4)	
2019	2,294 (21.3)	1,020 (46.1)	1,051 (74.5)	799 (15.7)	555 (15.7)	0 (0.0)	1,073 (17.8)	68 (14.6)	1,127 (17.2)	3,258 (14.1)	1,736 (13.9)	
CCI score												
Mean ± SD	0.36 (0.9)	0.38 (0.91)	0.45 (1.05)	0.39 (0.93)	0.42 (0.956)	0.33 (0.76)	0.41 (0.96)	0.38 (0.96)	0.43 (1.06)	0.58 (1.29)	0.83 (1.62)	
Hospitalized within the previous 12 months, *n* (%)												
Yes	5,526 (51.4)	750 (33.9)	662 (47.0)	3,044 (59.9)	1,900 (53.6)	172 (60.8)	2,859 (47.4)	215 (46.2)	1,794 (27.4)	4,020 (17.4)	2,746 (22.0)	
No	5,221 (48.6)	1,462 (66.1)	748 (53.0)	2,039 (40.1)	1,643 (46.4)	111 (39.2)	3,177 (52.6)	250 (53.8)	4,742 (72.6)	19,093 (82.6)	9,712 (78.0)	
Total person-years of follow-up												
Mean (SD)	2.2 (1.34)	1.02 (0.52)	0.68 (0.41)	2.58 (1.44)	2.62 (1.44)	3.61 (1.14)	2.56 (1.46)	2.67 (1.43)	2.62 (1.47)	2.62 (1.42)	2.6 (1.48)	
Comorbid psychiatric diagnoses												
Bipolar	1,129 (10.5)	152 (6.9)	270 (19.2)	743 (14.6)	329 (9.3)	34 (12.0)	663 (11.0)	42 (9.0)	509 (7.8)	1,537 (6.7)	1,178 (9.5)	
MDD	1,148 (10.7)	201 (9.1)	174 (12.3)	493 (9.7)	295 (8.3)	20 (7.1)	577 (9.6)	37 (8.0)	688 (10.5)	2,235 (9.7)	1,529 (12.3)	
M + O	1,954 (18.2)	318 (14.4)	300 (21.3)	845 (16.6)	523 (14.8)	37 (13.1)	1,065 (17.6)	70 (15.1)	1,162 (17.8)	3,806 (16.5)	2,475 (19.9)	
Dementia	73 (0.7)	10 (0.5)	15 (1.1)	48 (0.9)	28 (0.8)	≤3	52 (0.9)	≤3	79 (1.2)	927 (4.0)	442 (3.6)	
Suicide attempt	38 (0.4)	≤3	6 (0.4)	18 (0.4)	6 (0.2)	0 (0.0)	14 (0.2)	≤3	12 (0.2)	30 (0.1)	25 (0.2)	
Mental disorder due to drug use	445 (4.1)	42 (1.9)	33 (2.3)	320 (6.3)	255 (7.2)	30 (10.6)	278 (4.6)	34 (7.3)	178 (2.7)	642 (2.8)	390 (3.1)	

	Lurasidone (*n* = 812)	Zotepine (*n* = 637)	Aripiprazole (*n* = 6,188)	Ziprasidone (*n* = 164)	Quetiapine (*n* = 7,136)	Olanzapine (*n* = 2,014)	Flupentixol (*n* = 251)	Amisulpride (*n* = 1,120)	Clotiapine (*n* = 183)	Chlorpromazine (*n* = 167)	Sulpiride (*n* = 1,517)	
Gender, *n* (%)												
Male	341 (42.0)	351 (55.1)	2,716 (43.9)	74 (45.1)	3,453 (48.4)	1,067 (53.0)	131 (52.2)	611 (54.6)	113 (61.7)	110 (65.9)	730 (48.1)	
Female	471 (58.0)	286 (44.9)	3,472 (56.1)	90 (54.9)	3,683 (51.6)	947 (47.0)	120 (47.8)	509 (45.4)	70 (38.3)	57 (34.1)	787 (51.9)	
Age, years												
Mean ± SD	43.39 (14.51)	48.32 (12.58)	42.41 (15.29)	44.27 (13.21)	54.51 (17.4)	48.61 (14.1)	49.22 (12.5)	46.43 (13.59)	49.3 (12.72)	50.13 (11.65)	47.61 (15.03)	
Index year, *n* (%)												
2015	0 (0.0)	204 (32.0)	1,352 (21.8)	69 (42.1)	1,687 (23.6)	504 (25.0)	67 (26.7)	331 (29.6)	42 (23.0)	40 (24.0)	391 (25.8)	
2016	0 (0.0)	132 (20.7)	1,254 (20.3)	41 (25.0)	1,807 (25.3)	419 (20.8)	46 (18.3)	269 (24.0)	50 (27.3)	28 (16.8)	363 (23.9)	
2017	132 (16.3)	139 (21.8)	1,322 (21.4)	18 (11.0)	1,537 (21.5)	441 (21.9)	57 (22.7)	181 (16.2)	46 (25.1)	45 (26.9)	248 (16.3)^b^	
2018	272 (33.5)	102 (16.0)	1,172 (18.9)	18 (11.0)	1,157 (16.2)	351 (17.4)	40 (15.9)	169 (15.1)	19 (10.4)	35 (21.0)		
2019	408 (50.2)	60 (9.4)	1,088 (17.6)	18 (11.0)	948 (13.3)	299 (14.8)	41 (16.3)	170 (15.2)	26 (14.2)	19 (11.4)	230 (15.2)	
CCI Score												
Mean ± SD	0.38 (0.9)	0.61 (1.18)	0.41 (0.99)	0.38 (0.79)	0.93 (1.54)	0.5 (1.28)	0.51 (1.2)	0.36 (0.99)	0.52 (1.25)	0.93 (1.57)	0.46 (1.04)	
Hospitalized within the previous 12 months, *n* (%)												
Yes	93 (11.5)	114 (17.9)	551 (8.9)	8 (4.9)	306 (4.3)	162 (8.0)	23 (9.2)	58 (5.2)	13 (7.1)	10 (6.0)	26 (1.7)	
No	719 (88.5)	523 (82.1)	5,637 (91.1)	156 (95.1)	6,830 (95.7)	1,852 (92.0)	228 (90.8)	1,062 (94.8)	170 (92.9)	157 (94.0)	1,491 (98.3)	
Total person-years of follow-up												
Mean (SD)	1.09 (0.73)	2.8 (1.45)	2.53 (1.44)	3.17 (1.49)	2.61 (1.42)	2.63 (1.46)	2.67 (1.48)	2.8 (1.5)	2.61 (1.46)	2.52 (1.36)	2.68 (1.49)	
Comorbid psychiatric diagnoses												
Bipolar	115 (14.2)	61 (9.6)	402 (6.5)	20 (12.2)	460 (6.5)	49 (2.4)	6 (2.4)	27 (2.4)	6 (3.3)	5 (3.0)	12 (0.8)	
MDD	144 (17.7)	99 (15.5)	737 (11.9)	21 (12.8)	864 (12.1)	132 (6.6)	20 (8.0)	37 (3.3)	16 (8.7)	10 (6.0)	68 (4.5)	
M + O	222 (27.3)	153 (24.0)	1,233 (19.9)	39 (23.8)	1,516 (21.2)	256 (12.7)	33 (13.2)	81 (7.2)	27 (14.8)	23 (13.8)	144 (9.5)	
Dementia	8 (1.0)	11 (1.7)	97 (1.6)	≤3	393 (5.5)	34 (1.7)	≤3	5 (0.5)	≤3	<−3	16 (1.1)	
Suicide attempt	≤3	≤3	6 (0.1)	0 (0.0)	6 (0.1)	≤3	0 (0.0)	0 (0.0)	0 (0.0)	0 (0.0)	0 (0.0)	
Mental disorder due to drug use	24 (3.0)	26 (4.1)	80 (1.3)	6 (3.7)	150 (2.1)	31 (1.5)	5 (2.0)	16 (1.4)	14 (7.7)	4 (2.4)	6 (0.4)	

*Abbreviations*: CCI, Charlson Comorbidity Index; M + O, major depressive disorder and other affective mood disorders; MMD, major depressive disorder; PP1M, paliperidone administered monthly; PP3M, paliperidone administered 3-monthly; SD, standard deviation.

a Fewer than 50 eligible patients received oral clozapine, loxapine, pimozide, thioridazine, chlorprothixene, methotrimeprazine, trifluoperazine, and zuclopenthixol and are not presented.

b To protect patient privacy, all nonzero counts that were less than 4 were not shown.

The proportion of psychiatric comorbidities was high. Bipolar disorder and major depressive disorder were present most frequently in patients in the oral aripiprazole, oral lurasidone, and oral zotepine exposure groups; dementia was most frequent in the oral quetiapine, risperidone, and haloperidol groups; and mental disorders linked to illicit drug use were highest in the LAI haloperidol, LAI clopenthixol, and LAI fluphenazine groups (Table 1).

There were a total of 228,791.08 person-years of follow-up in the active treatment cohort, and the mean follow-up period per patient was 2.48 person-years (standard deviation [SD] 1.46). The mean (SD) lengths of follow-up were 2.2 years (1.34) in the PP1M group and 1.02 (0.52) years in the PP3M group.

### All-cause death rate

The crude all-cause death rates during the active treatment period were 7.40 per 1,000 person-years (95% confidence interval [CI] 5.94–9.11) in the PP1M exposure group, 9.93 per 1,000 person-years (95% CI 5.88–15.79) in the PP3M group, 7.10 per 1,000 person-years (95% CI 2.26–17.12) in the aripiprazole LAI group, and 6.69 per 1,000 person-years (95% CI 4.46–9.66) in the risperidone LAI group (Table 2). The crude all-cause death rates ranged from 2.43 to 25.67 per 1,000 person-years in users of typical LAIs, and from 6.62 to 31.93 per 1,000 person-years in users of oral antipsychotics.

**Table 2.** Incidence (per 1,000 person-years) of all-cause mortality and completed suicide (active treatment period).

	Total no. of new AP users	Total person-years	No. of all-cause deaths	Incidence of all-cause deaths (95% CI)	No. of completed suicides	Incidence of completed suicide (95% CI)
Atypical LAI						
PP1M	10,747	11,355.47	84	7.40 (5.94, 9.11)	23	2.03 (1.32, 2.99)
PP3M	2,212	1,610.61	16	9.93 (5.88, 15.79)	5	3.10 (1.14, 6.88)
Aripiprazole	1,410	563.45	4	7.10 (2.26, 17.12)	0	–
Risperidone	5,083	3,887.08	26	6.69 (4.46, 9.66)	7	1.80 (0.79, 3.56)
Typical LAI						
Haloperidol	3,543	3,094.72	18	5.82 (3.56, 9.01)	2	0.64 (0.11, 2.14)
Clopenthixol	283	116.85	3	25.67 (6.53, 69.87)	1	8.56 (0.43, 42.21)
Flupentixol	6,036	5,383.43	27	5.02 (3.37, 7.20)	9	1.67 (0.82, 3.07)
Fluphenazine	465	412.44	1	2.43 (0.12, 11.96)	0	–
Oral AP						
Paliperidone	6,536	5,775.43	46	7.97 (5.90, 10.53)	15	2.60 (1.51, 4.19)
Risperidone	23,113	25,495.94	325	12.75 (11.42, 14.19)	52	2.04 (1.54, 2.65)
Haloperidol	12,458	9,099.32	124	13.63 (11.38, 16.19)	6	0.66 (0.27, 1.37)
Lurasidone	812	411.86	5	12.14 (4.45, 26.91)	1	2.42 (0.12, 11.97)
Zotepine	637	564.33	17	30.12 (18.13, 47.25)	1	1.77 (0.09, 8.74)
Aripiprazole	6,188	7,413.75	62	8.36 (6.47, 10.65)	10	1.35 (0.69, 2.40)
Ziprasidone	164	194.01	2	10.31 (1.73, 34.06)	0	–
Quetiapine	7,136	8,794.77	220	25.01 (21.87, 28.49)	12	1.36 (0.74, 2.32)
Olanzapine	2,014	2,734.78	38	13.90 (9.97, 18.88)	5	1.83 (0.67, 4.05)
Flupentixol	251	301.94	2	6.62 (1.11, 21.88)	0	–
Amisulpride	1,120	1,684.1	19	11.28 (6.99, 17.29)	1	0.59 (0.03, 2.93)
Clotiapine	183	187.94	6	31.93 (12.94, 66.40)	0	–
Chlorpromazine	167	186.17	2	10.74 (1.80, 35.49)	0	–
Sulpiride	1,517	1,637.56	11	6.72 (3.53, 11.68)	0	–

*Note*: There were no deaths found in the antipsychotic exposure groups of oral pimozide, trifluoperazine, and methotrimeprazine.

*Abbreviations*: AP, antipsychotic; CI, confidence interval; LAI, long-acting injection; PP1M, paliperidone administered monthly; PP3M, paliperidone administered 3-monthly.

The cumulative incidences of all-cause death in the PP1M and PP3M groups per 1,000 patients were 2.23 and 0.90, respectively, at 30 days, 7.91 and 7.23 at 90 days, and 12.56 and 11.75 at 180 days. At 180 days, the cumulative incidences of all-cause death ranged from 13.92 to 24.73 per 1,000 patients for typical LAIs, and from 9.23 to 49.18 per 1,000 patients for oral antipsychotics (Table 3).

**Table 3.** Cumulative incidences of all-cause death and completed suicide up to 30, 90, and 180 days (per 1,000 patients) after the index date in new users^a^ of LAI antipsychotics (fixed cohort analysis).

	Total no. of antipsychotic users	No. within 30 days	30-day cumulative incidence	No. within 90 days	90-day cumulative incidence	No. within 180 days	180-day cumulative incidence
*All-cause mortality*							
Atypical long-acting injectable antipsychotic							
PP1M	10,747	24	2.23	85	7.91	135	12.56
PP3M	2,212	2	0.90	16	7.23	26	11.75
Aripiprazole	1,410	2	1.42	6	4.26	8	5.67
Risperidone	5,083	14	2.75	55	10.82	88	17.31
Typical long-acting injectable antipsychotic								
Haloperidol	3,543	18	5.08	47	13.27	70	19.76
Clopenthixol	283	0	0.00	5	17.67	7	24.73
Flupentixol	6,036	22	3.64	58	9.61	84	13.92
Fluphenazine	465	3	6.45	5	10.75	8	17.20
Oral AP							
Paliperidone	6,536	25	3.82	42	6.43	67	10.25
Risperidone	23,113	141	6.10	302	13.07	503	21.76
Haloperidol	12,458	267	21.43	435	34.92	592	47.52
Lurasidone	812	2	2.46	4	4.93	13	16.01
Zotepine	637	5	7.85	11	17.27	22	34.54
Aripiprazole	6,188	18	2.91	49	7.92	77	12.44
Ziprasidone	164	2	12.20	2	12.20	4	24.39
Quetiapine	7,136	68	9.53	164	22.98	240	33.63
Olanzapine	2,014	12	5.96	33	16.39	46	22.84
Flupentixol	251	1	3.98	3	11.95	4	15.94
Amisulpride	1,120	4	3.57	13	11.61	20	17.86
Clotiapine	183	6	32.79	7	38.25	9	49.18
Chlorpromazine	167	3	17.96	5	29.94	6	35.93
Sulpiride	1,517	4	2.64	5	3.30	14	9.23
*Completed suicide*							
Atypical long-acting injectable antipsychotic							
PP1M	10,747	3	0.28	26	2.42	44	4.09
PP3M	2,212	0	0.00	4	1.81	7	3.16
Aripiprazole	1,410	0	0.00	3	2.13	4	2.84
Risperidone	5,083	2	0.39	15	2.95	22	4.33
Typical long-acting injectable antipsychotic							
Haloperidol	3,543	1	0.28	9	2.54	12	3.39
Clopenthixol	283	0	0.00	2	7.07	2	7.07
Flupentixol	6,036	7	1.16	17	2.82	22	3.64
Oral AP							
Paliperidone	6,536	6	0.92	10	1.53	17	2.60
Risperidone	23,113	17	0.74	34	1.47	62	2.68
Haloperidol	12,458	16	1.28	26	2.09	41	3.29
Lurasidone	812	1	1.23	2	2.46	5	6.16
Zotepine	637	0	0.00	1	1.57	3	4.71
Aripiprazole	6,188	3	0.48	8	1.29	15	2.42
Ziprasidone	164	0	0.00	0	0.00	2	12.20
Quetiapine	7,136	8	1.12	13	1.82	16	2.24
Olanzapine	2,014	0	0.00	2	0.99	3	1.49
Flupentixol	251	0	0.00	1	3.98	1	3.98
Amisulpride	1,120	0	0.00	1	0.89	2	1.79
Clotiapine	183	0	0.00	1	5.46	1	5.46

*Abbreviations*: LAI, long-acting injection; PP1M, paliperidone administered monthly; PP3M, paliperidone administered 3-monthly.

a A new user is a prevalent case who has not received the index antipsychotic within the last 12 months.

#### Comparative analysis of the relative risk of all-cause death

**Figure 2.**
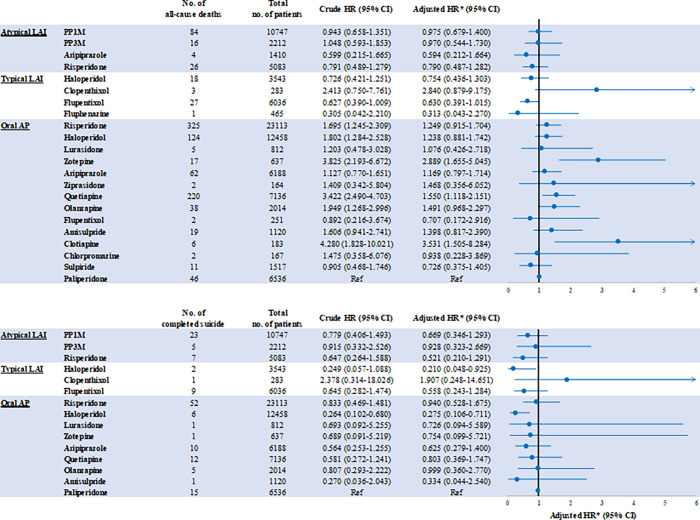
Crude and adjusted^a^ hazard ratios of (A) all-cause death and (B) completed suicide in all antipsychotic users compared with oral paliperidone using a Cox regression model. ^a^Adjusted for age (as a category variable), gender, Charlson Comorbidity Index score, hospitalization (as a category variable), comorbid bipolar disorder, major depressive disorder, or other affective mood disorders, mental disorder due to drug use, and index year.

The crude HR estimates for the risk of all-cause death compared to oral paliperidone were <1 for all atypical LAIs except PP3M (HR 1.05), and for all typical LAIs except clopenthixol (HR 2.413; Figure 2). Age, sex, CCI score, index year, inpatient episodes in the 12-month baseline period, and co-psychiatric comorbidities were identified as confounding factors and included in the regression model. In the adjusted analysis, the adjusted hazard ratios (aHRs) for the risk of all-cause death compared to oral paliperidone were <1 for all atypical LAIs and for all typical LAIs except clopenthixol. There was no statistically significant increase in the risk of all-cause death during the active treatment period in any of the atypical or typical LAI groups compared with oral paliperidone. The aHRs for the risk of all-cause death compared with oral paliperidone were 0.98 (95% CI 0.68–1.40) for PP1M and 0.97 (95% CI 0.54–1.73) for PP3M.

For oral antipsychotics, the risk of all-cause death was statistically significantly increased compared with oral paliperidone in users of oral risperidone, oral haloperidol, zotepine, quetiapine, olanzapine, and clotiapine in the crude analysis, but the risk only remained statistically significant for zotepine, quetiapine, and clotiapine in the adjusted analysis (aHRs 2.89 [95% CI 1.66–5.05], 1.55 [95% CI 1.12–2.15], and 3.53 [95% CI 1.51–8.28], respectively; Figure 2).

### Rate of completed suicide

The rates of completed suicide during the active treatment period were 2.03 per 1,000 person-years (95% CI 1.32–2.99) in the PP1M exposure group, 3.10 per 1,000 person-years (95% CI 1.14–6.88) in the PP3M group, and 1.80 per 1,000 person-years (95% CI 0.79–3.56) in the risperidone LAI group (Table 2). The rates of completed suicide ranged from 0.64 to 8.56 per 1,000 person-years in users of typical LAIs, and from 0.59 to 2.60 per 1,000 person-years in users of oral antipsychotics.

The cumulative incidences of completed suicide in the PP1M and PP3M groups were 0.28 per 1,000 and 0 per 1,000 patients, respectively, at 30 days, 2.42 per 1,000 and 1.81 per 1,000 patients at 90 days, and 4.09 per 1,000 and 3.16 per 1,000 patients at 180 days. At 180 days, the cumulative incidences of completed suicide ranged from 3.39 per 1,000 to 7.07 per 1,000 patients for typical LAIs, and from 1.49 to 12.20 per 1,000 for oral antipsychotics (Table 3).

#### Comparative analysis of the relative risk of completed suicide

There was no statistically significant increase in the risk of completed suicide during the active treatment period in users of any of the atypical or typical LAIs compared with oral paliperidone (crude and adjusted analyses; Figure 2). There was a statistically significantly lower risk of completed suicide for haloperidol (LAI and oral forms), compared with oral paliperidone (aHRs 0.21 [95% CI 0.05–0.93] and 0.28 [95% CI 0.11–0.71], respectively). The aHRs for the risk of completed suicide compared with oral paliperidone were 0.67 (95% CI 0.35–1.29) for PP1M and 0.93 (95% CI 0.32–2.67) for PP3M. The aHR estimates were all <1 for all atypical and typical LAIs except clopenthixol, and for all oral antipsychotics.

### Sensitivity analysis

The sensitivity analysis included a total of 62% (*n* = 99,739) of the enrolled cohort who held a catastrophic illness certificate for schizophrenia/schizoaffective disorder. Patients holding a certificate were older (mean age 46.9 vs. 43.9 years; *p* < 0.001), and were less likely to be female (47.4 vs. 50.1%; *p* < 0.001) than patients without a certificate.

The aHRs for the risk of all-cause death compared with oral paliperidone were 0.88 (95% CI 0.58–1.35) for PP1M and 1.07 (95% CI 0.56–2.04) for PP3M. The aHRs for the risk of completed suicide compared with oral paliperidone were 0.83 (95% CI 0.34–2.03) for PP1M and 1.40 (95% CI 0.37–5.22) for PP3M (Figure S2).

In contrast to the main analysis, there was a decreased risk of death in users of LAI flupentixol and an increased risk of all-cause death in users of zotepine and quetiapine compared with oral paliperidone (Figure S2). There was no statistically significant difference in the risk of completed suicide in users of oral and LAI haloperidol compared to oral paliperidone in the sensitivity analysis.

### Overall mortality rate in patients with schizophrenia

Crude annual mortality rates in patients with confirmed schizophrenia/schizoaffective disorder were more than double those of the general population in Taiwan over the study years and increased from 1,559 per 100,000 population in 2015 to 1950 per 100,000 population in 2019 (25% increase). Crude annual mortality rates also appeared to increase in the general population (by 6.6%), from 697 per 100,000 population in 2015 to 743 per 100,000 in 2019 (Figure S3). Deaths per 100,000 population were higher in men and increased with age in both populations but were higher in patients with schizophrenia/schizoaffective disorder than the general population of Taiwan in all age groups (Figure S4).

## Discussion

Patients with schizophrenia/schizoaffective disorder have a higher risk of death and die at a younger age than the general population. Yet few population-based studies conducted across the full spectrum of available antipsychotics have assessed the risk of death in patients with these disorders. To the best of our knowledge, ours is the first evaluation of mortality in a large cohort of patients with schizophrenia/schizoaffective disorder using PP3M, and one of few to compare the relative risks of death between individual LAIs and oral antipsychotics.

The risks of all-cause death and completed suicide were not increased in patients receiving LAIs compared to oral paliperidone. The results were similar for atypical and typical LAIs and were confirmed in a sensitivity analysis of patients with an established diagnosis of schizophrenia/schizoaffective disorder. LAI and oral haloperidol were associated with a statistically significantly lower risk of completed suicide compared to oral paliperidone in the main analysis, but this finding was not present in the sensitivity analysis of patients with confirmed schizophrenia/schizoaffective disorder registered in the RCI. There was an isolated finding of a statistically significantly lower risk of all-cause death in users of LAI flupentixol compared to oral paliperidone in the sensitivity analysis. No other differences between LAI and oral antipsychotics compared to oral paliperidone were observed in the both the main analysis and the sensitivity analysis.

The apparent reduction in completed suicide but not all-cause death in users of LAI or oral haloperidol compared to oral paliperidone may reflect characteristics of the population prescribed these antipsychotics. Compared with oral paliperidone, patients receiving oral haloperidol were older, had a higher CCI, and had higher frequencies of psychiatric comorbidities including dementia. Patients using LAI haloperidol were also older than patients who received oral paliperidone, were more likely to be male, but had a similar CCI and similar or lower rate of psychiatric comorbidities, with the exception of a lower prevalence of mental disorders due to drug use.

The hierarchical new user design of our study means that the period of follow-up was longer in patients who commenced and remained on one antipsychotic during the study period than the patients who switched to LAI antipsychotics, suggesting a stable or well-controlled disease. Patients with symptoms or side effects necessitating a change in their antipsychotic during the study were therefore enrolled elsewhere in the treatment hierarchy, and the possible follow-up period was reduced. As a result of this design, the demographic and disease features of patients differed across the exposure groups. Patients prescribed the newer LAI PP1M or PP3M had short follow-up periods (2.2 and 1.02 years, respectively), reflecting their recent availability on the market, and that they may have been initiated in patients whose responses to other therapies used during the study period were suboptimal. Patients in the PP1M and PP3M groups had high rates of psychiatric hospital admission in the 12 months prior to the index date, consistent with either severe disease, or with the current practice for PP1M and PP3M to be initiated while as an inpatient. Patients who received the older LAI clopenthixol had a longer follow-up period (mean 3.61 years), suggesting a more stable group who remained on this drugs for long periods.

The limitation of the hierarchical approach is that patients initiated on oral treatments who responded well and remained stable may have had a lower risk of death or completed suicide than those patients who were switched to an LAI antipsychotic. Despite this limitation, we did not observe a higher risk of death or completed suicide in users of PP3M, or PP1M in comparison with oral paliperidone.

The sensitivity analysis using the RCI included 62% of patients with a schizophrenia/schizoaffective disorder in the NHIRD. Patients without a certificate were younger and included more women than patients who were in the RCI, possibly reflecting social sensitivity associated with a schizophrenia/schizoaffective diagnosis. Oral antipsychotics are not costly in Taiwan, and the incentive for financial support through the RCI may be low for patients using these drugs and who are treated as outpatients. Patients with severe disease diagnosed during hospitalization would receive a catastrophic illness card, which is easily obtained during hospitalization and would cover all hospitalization costs associated with schizophrenia.

Few studies to date have assessed potential associations between individual LAIs and mortality risk. A nationwide cohort study in Sweden found that compared with nonuse, mortality risk was lowest for prevalent patients taking second-generation LAIs, being lowest for PP1M (aHR 0.11 [95% CI 0.03–0.43] compared to no antipsychotic use) [20]. The incident (new user) cohort was too small to allow meaningful comparisons. The meta-analysis of clinical data from randomized trials by Kishi *et al*. [15] found no significant difference between pooled LAIs and pooled oral antipsychotics in terms of all-cause mortality or completed suicide. Our larger study using real-world data allowed estimation of the risk of death associated with individual LAIs compared to a commonly used atypical oral antipsychotic. The results showed no increased risk of all-cause death or completed suicide for any LAI, including PP1M and the newest atypical LAI, PP3M compared to oral paliperidone and other LAI.

The major strength of our study is the completeness and accuracy of data capture in terms of antipsychotic treatments and outcome. The NHIRD captures complete treatment information regardless of where it is prescribed (outpatient, inpatient, and pharmacy dispensing data), allowing accurate classification of antipsychotic exposure. Detailed information on demography, comorbidities and current medications, hospitalization, and services rendered by healthcare providers in Taiwan allows identification of potential confounding factors. Reporting of deaths to the Death Registry is mandatory, and the Registry captures all deaths that occur in and out of hospital. The sensitivity analysis using the RCI verified the results in a patient population in whom the diagnosis can be considered confirmed. Another strength is that the population-based study allowed us to estimate mortality for individual drugs.

Potential limitations include a lack of clinical information in the NHIRD about disease severity, progression or treatment response, and potential associations with mortality. The study was designed to capture the period of PP3M introduction in Taiwan and the available period of follow-up after the index date was limited. Consequently, all-cause death data and completed suicide data in the PP3M exposure group are not yet robust due to the relatively small number of new users and limited follow-up period available. Confounding by indication is major challenge for retrospective claims-based studies. While we did our best to account for potential confounding in our study, the possibility of residual confounding cannot be completely excluded. Cumulative exposure to antipsychotics that may impact mortality rates were not captured in our study. Finally, drug adherence is one of the major determinants of prognosis in schizophrenia with treatment gaps associated with relapse, hospitalization, suicide attempts, and mortality [12,21,22]. The measurement of adherence is difficult [23], and the NHIRD claims database is unable to capture short treatment gaps, particularly for users of oral antipsychotic, and we were unable to adjust for nonadherence in our analysis. In this regard, LAIs are gaining traction as a means to improve adherence, and while previously reserved for the most challenging patients, are being increasingly considered as initial treatment in any patient [23,24].

In conclusion, the risks of all-cause death and completed suicide were not increased in patients initiated with atypical LAI antipsychotics in comparison with oral paliperidone. These data confirm the safety of LAIs and provide reassurance to patients and physicians.

## Data Availability

The data underlying this study are from the National Health Insurance Research Database which has been transferred to the Health and Welfare Data Science Center (HWDC). The Taiwan government prohibits the release of the NHI claims dataset to the public domain. Interested researchers can obtain the data through formal application to the HWDC, Department of Statistics, Ministry of Health and Welfare, Taiwan (http://dep.mohw.gov.tw/DOS/np-2497-113.html).

## Supplementary Materials

To view supplementary material for this article, please visit http://doi.org/10.1192/j.eurpsy.2021.2258. click here to view supplementary material
