# Supplementary material for: Risks of all-cause death and completed suicide in patients with schizophrenia/schizoaffective disorder treated with long-acting injectable or oral antipsychotics: A population-based retrospective cohort study in Taiwan
Source: Eur Psychiatry. 2021 Dec 13;65(1):e5. doi: 10.1192/j.eurpsy.2021.2258 (PMC8853856; doi:10.1192/j.eurpsy.2021.2258)
Supplement: Supplementary file 1 [file S0924933821022586sup001.docx]

**Risks of all-cause death and completed suicide in patients with schizophrenia/schizoaffective disorder treated with long-acting injectable or oral antipsychotics: a population-based retrospective cohort study in Taiwan**

Chao-Hsiun Tang, Shih-Pei Shen, Min-Wei Huang_,_ Hong Qiu, Sayuri Watanabe, Choo Hua Goh, Yanfang Liu

**SUPPLEMENT**

**Schematic of the hierarchical enrollment approach.**

Each patient’s record was first checked to see if they had received a prescription for PP3M,

=> If yes, the patient was enrolled as a new user of PP3M,

=> If no, the record was then checked for a prescription for PP1M,

=> If yes, the patient was enrolled as a new user of PP1M,

=> If no, the record was then checked for a prescription for risperidone LAI,

=> If yes, the patient was enrolled as a new user of risperidone LAI,

=> If no, the record was then checked for a prescription for the next typical LAI,

=> If yes, the patient was enrolled as a new user of that typical LAI,

=> If no, the record was then checked for a prescription for oral paliperidone,

=> If yes, the patient was enrolled as a new user of oral paliperidone

=> If no, the record was then checked for a prescription for the first of the other oral antipsychotics,

=> And so on.

**Table S1 X60-X84, Y87.0  Intentional self-harm**

| **X60** | Intentional self-poisoning by and exposure to nonopioid analgesics, antipyretics and antirheumatics |
| --- | --- |
| **X61** | Intentional self-poisoning by and exposure to antiepileptic, sedative-hypnotic, anti-parkinsonism and psychotropic drugs, not elsewhere classified |
| **X62** | Intentional self-poisoning by and exposure to narcotics and psychodysleptics [hallucinogens], not elsewhere classified |
| **X63** | Intentional self-poisoning by and exposure to other drugs acting on the autonomic nervous system |
| **X64** | Intentional self-poisoning by and exposure to other and unspecified drugs, medicaments and biological substances |
| **X65** | Intentional self-poisoning by and exposure to alcohol |
| **X66** | Intentional self-poisoning by and exposure to organic solvents and halogenated hydrocarbons and their vapours |
| **X67** | Intentional self-poisoning by and exposure to carbon monoxide and other gases and vapours |
| **X68** | Intentional self-poisoning by and exposure to pesticides |
| **X69** | Intentional self-poisoning by and exposure to other and unspecified chemicals and noxious substances |
| **X70** | Intentional self-harm by hanging, strangulation and suffocation |
| **X71** | Intentional self-harm by drowning and submersion |
| **X72** | Intentional self-harm by handgun discharge |
| **X73** | Intentional self-harm by rifle, shotgun and larger firearm discharge |
| **X74** | Intentional self-harm by other and unspecified firearm discharge |
| **X75** | Intentional self-harm by explosive material |
| **X76** | Intentional self-harm by smoke, fire and flames |
| **X77** | Intentional self-harm by steam, hot vapours and hot objects |
| **X78** | Intentional self-harm by sharp object |
| **X79** | Intentional self-harm by blunt object |
| **X80** | Intentional self-harm by jumping from a high place |
| **X81** | Intentional self-harm by jumping or lying before moving object |
| **X82** | Intentional self-harm by crashing of motor vehicle |
| **X83** | Intentional self-harm by other specified means |
| **X84** | Intentional self-harm by unspecified means |
| **Y87.0** | Sequelae of intentional self-harm |

**Table S2** Additional demographic characteristics of the patient cohort by treatment assignment**

|  | **Long-acting injectable antipsychotic** | | | | | | | | | | | | | | | **Oral antipsychotic→** | | |
| --- | --- | --- | --- | --- | --- | --- | --- | --- | --- | --- | --- | --- | --- | --- | --- | --- | --- | --- |
|  | **PP1M  (n=10747)** | **PP3M (n=2212)** | | **Aripiprazole (n=1410)** | | **Risperidone  (n=5083)** | | **Haloperidol (n=3543)** | | **Clopenthixol (n=283)** | | **Flupentixol (n=6036)** | | **Fluphenazine (n=465)** | | **Paliperidone**  **(n =6536)** | **Risperidone (n=23,113)** | **Haloperidol (n=12,458)** |
| **Age, years** n (%) | | |  | |  | |  | |  | |  | |  | |  |  |  |  |
| Median | 42.99 | 44.06 | | 40.37 | | 44.4 | | 44.88 | | 45.34 | | 45.13 | | 43.21 | | 41.46 | 45.77 | 48.35 |
| 18 to 19 | 138 (1.3) | 15 (0.7) | | 25 (1.8) | | 54 (1.1) | | 18 (0.5) | | 16 (5.7)* | | 61 (1.0) | | 63 (13.5)* | | 171 (2.6) | 475 (2.1) | 129 (1.0) |
| 20 to 29 | 1478 (13.8) | 278 (12.6) | | 285 (20.2) | | 635 (12.5) | | 309 (8.7) | |  |  | 595 (9.9) | |  |  | 1201 (18.4) | 3404 (14.7) | 1113 (8.9) |
| 30 to 39 | 2785 (25.9) | 530 (24.0) | | 369 (26.2) | | 1197 (23.5) | | 872 (24.6) | | 74 (26.1) | | 1420 (23.5) | | 106 (22.8) | | 1639 (25.1) | 4861 (21.0) | 2421 (19.4) |
| 40 to 49 | 3002 (27.9) | 688 (31.1) | | 350 (24.8) | | 1429 (28.1) | | 1125 (31.8) | | 100 (35.3) | | 1877 (31.1) | | 151 (32.5) | | 1557 (23.8) | 4846 (21.0) | 3114 (25.0) |
| 50 to 59 | 2299 (21.4) | 512 (23.1) | | 247 (17.5) | | 1165 (22.9) | | 905 (25.5) | | 79 (27.9) | | 1416 (23.5) | | 89 (19.1) | | 1184 (18.1) | 4741 (20.5) | 2950 (23.7) |
| 60 to 69 | 895 (8.3) | 173 (7.8) | | 123 (8.7) | | 479 (9.4) | | 280 (7.9) | | 14 (4.9)* | | 578 (9.6) | | 49 (10.5) | | 607 (9.3) | 2778 (12.0) | 1664 (13.4) |
| 70 to 79 | 139 (1.3) | 13 (0.6) | | 11 (0.8) | | 101 (2.0) | | 29 (0.8) | |  |  | 81 (1.3) | | 7 (1.5)* | | 149 (2.3) | 1188 (5.1) | 625 (5.0) |
| ≥ 80 | 11 (0.1) | 3 (0.1) | | 0 (0.0) | | 23 (0.5) | | 5 (0.1) | | 0 (0.0) | | 8 (0.1) | |  |  | 28 (0.4) | 820 (3.5) | 442 (3.5) |
| **CCI score** n (%) | |  | |  | |  | |  | |  | |  | |  | |  |  |  |
| CCI=0 | 8385 (78.0) | 1701 (76.9) | | 1054 (74.8) | | 3878 (76.3) | | 2643 (74.6) | | 222 (78.4) | | 4560 (75.5) | | 362 (77.8) | | 5023 (76.9) | 16747 (72.5) | 7973 (64.0) |
| CCI=1 | 1548 (14.4) | 336 (15.2) | | 217 (15.4) | | 795 (15.6) | | 601 (17.0) | | 42 (14.8) | | 938 (15.5) | | 68 (14.6) | | 898 (13.7) | 3207 (13.9) | 2135 (17.1) |
| CCI=2 | 504 (4.7) | 106 (4.8) | | 80 (5.7) | | 237 (4.7) | | 176 (5.0) | | 10 (3.5) | | 313 (5.2) | | 15 (3.2) | | 312 (4.8) | 1530 (6.6) | 1049 (8.4) |
| CCI≥3 | 310 (2.9) | 69 (3.1) | | 59 (4.2) | | 173 (3.4) | | 123 (3.5) | | 9 (3.2) | | 225 (3.7) | | 20 (4.3) | | 303 (4.6) | 1629 (7.0) | 1301 (10.4) |
|  | **Lurasidone (n=812)** | **Zotepine (n=637)** | | **Aripiprazole (n=6188)** | | **Ziprasidone (n=164)** | | **Quetiapine (n=7136)** | | **Olanzapine (n=2014)** | | **Flupentixol (n=251)** | | **Amisulpride (n=1120)** | | **Clotiapine (n=183)** | **Chlorpromazine (n=167)** | **Sulpiride (n=1517)** |
| **Age, years** n (%) | |  | |  | |  | |  | |  | |  | |  | |  |  |  |
| Median | 43.02 | 48.47 | | 40.53 | | 42.99 | | 53.88 | | 49.08 | | 49.96 | | 46.97 | | 50.17 | 50.93 | 47.62 |
| 18 to 19 | 27 (3.3) | 3 (0.5) | | 234 (3.8) | | 23 (14.0)* | | 68 (1.0) | | 24 (1.2) | | 20 (8.0)* | | 16 (1.4) | | 11 (6.0)* | 0 (0.0) | 21 (1.4) |
| 20 to 29 | 150 (18.5) | 52 (8.2) | | 1270 (20.5) | |  |  | 512 (7.2) | | 186 (9.2) | |  |  | 127 (11.3) | |  | 10 (6.0) | 186 (12.3) |
| 30 to 39 | 154 (19.0) | 113 (17.7) | | 1492 (24.1) | | 45 (27.4) | | 1030 (14.4) | | 366 (18.2) | | 41 (16.3) | | 232 (20.7) | | 35 (19.1) | 21 (12.6) | 287 (18.9) |
| 40 to 49 | 196 (24.1) | 178 (27.9) | | 1282 (20.7) | | 40 (24.4) | | 1336 (18.7) | | 486 (24.1) | | 65 (25.9) | | 270 (24.1) | | 45 (24.6) | 46 (27.5) | 346 (22.8) |
| 50 to 59 | 174 (21.4) | 178 (27.9) | | 1034 (16.7) | | 35 (21.3) | | 1551 (21.7) | | 529 (26.3) | | 83 (33.1) | | 298 (26.6) | | 54 (29.5) | 59 (35.3) | 368 (24.3) |
| 60 to 69 | 85 (10.5) | 90 (14.1) | | 586 (9.5) | | 16 (9.8) | | 1262 (17.7) | | 303 (15.0) | | 32 (12.7) | | 139 (12.4) | | 30 (16.4) | 25 (15.0) | 212 (14.0) |
| 70 to 79 | 23 (2.8) | 17 (2.7) | | 216 (3.5) | | 5 (3.0) | | 707 (9.9) | | 88 (4.4) | | 10 (4.0)* | | 32 (2.9) | | 8 (4.4)* | 6 (3.6) | 62 (4.1) |
| ≥ 80 | 3 (0.4) | 6 (0.9) | | 74 (1.2) | | 0 (0.0) | | 670 (9.4) | | 32 (1.6) | |  |  | 6 (0.5) | |  | 0 (0.0) | 35 (2.3) |
| **CCI score** n (%) | |  | |  | |  | |  | |  | |  | |  | |  |  |  |
| CCI=0 | 630 (77.6) | 430 (67.5) | | 4767 (77.0) | | 126 (76.8) | | 4164 (58.4) | | 1539 (76.4) | | 188 (74.9) | | 901 (80.4) | | 129 (70.5) | 98 (58.7) | 1128 (74.4) |
| CCI=1 | 110 (13.5) | 122 (19.2) | | 832 (13.4) | | 19 (11.6) | | 1327 (18.6) | | 268 (13.3) | | 32 (12.7) | | 134 (12.0) | | 34 (18.6) | 33 (19.8) | 226 (14.9) |
| CCI=2 | 44 (5.4) | 38 (6.0) | | 342 (5.5) | | 14 (8.5) | | 768 (10.8) | | 91 (4.5) | | 17 (6.8) | | 51 (4.6) | | 14 (7.7) | 15 (9.0) | 98 (6.5) |
| CCI≥3 | 28 (3.4) | 47 (7.4) | | 247 (4.0) | | 5 (3.0) | | 877 (12.3) | | 116 (5.8) | | 14 (5.6) | | 34 (3.0) | | 6 (3.3) | 21 (12.6) | 65 (4.3) |

PP1M: paliperidone monthly; PP3M: paliperidone three-monthly; CCI: Charlson Comorbidity Index; SD, standard deviation

* To protect patient privacy, all non-zero counts that were less than four were grouped with an adjacent category

**Fewer than 50 eligible patients received oral clozapine, loxapine, pimozide, thioridazine, chlorprothixene, methotrimeprazine, trifluoperazine, and zuclopenthixol and are not presented.


PP1M: paliperidone monthly; PP3M: paliperidone three-monthly; CCI: Charlson Comorbidity Index; SD, standard deviation

* To protect patient privacy, all non-zero counts that were less than four were grouped with an adjacent category

**Fewer than 50 eligible patients received oral clozapine, loxapine, pimozide, thioridazine, chlorprothixene, methotrimeprazine, trifluoperazine, and zuclopenthixol and are not presented.

**Figure S1** Patient enrollment diagram (oral antipsychotics)

* To protect patient privacy, all non-zero counts that were less than four were suppressed and grouped with an adjacent category

**Figure S2** Crude and adjusted hazard ratios* of all-cause death and completed suicide in all antipsychotic users in all antipsychotic users - Registry of Catastrophic Illness, sensitivity analysis

*adjusted for age (as a category variable), gender, Charlson Co-morbidity Index score, hospitalization (as a category variable), comorbid bipolar disorder, major depressive disorder or other mood disorder, mental disorder due to drug use, and index year

**Figure S3** Mortality rate per 100,000 population in the general population and in patients with schizophrenia/schizoaffective disorder in Taiwan from 2015 to 2019 (Data source: Ministry of Health and Welfare, Taiwan)


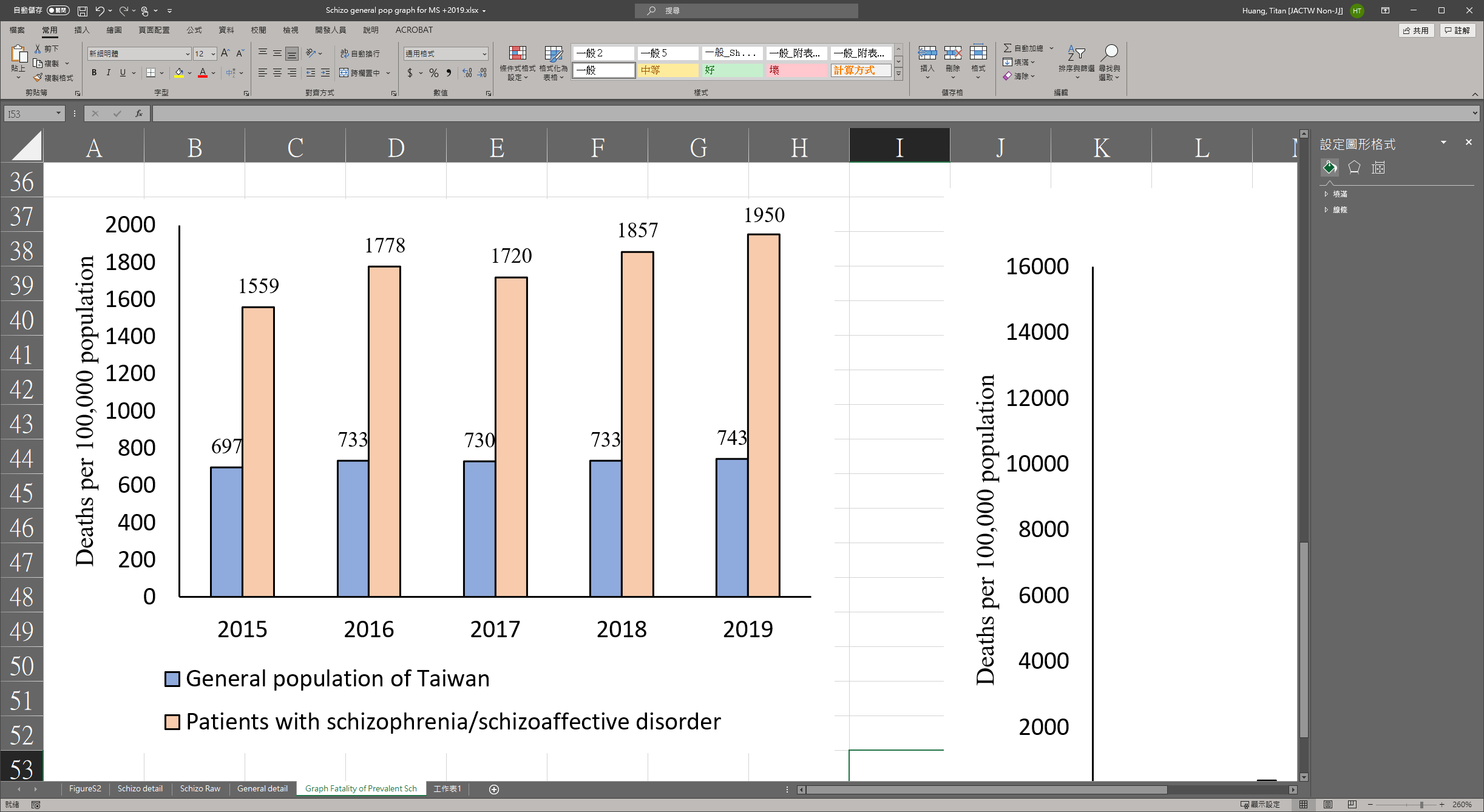


**Figure S4** Mortality rate per 100,000 population in the general population and in patients with schizophrenia/schizoaffective disorder in Taiwan from 2015 to 2019 by age group and gender (Data source: Ministry of Health and Welfare, Taiwan)

**
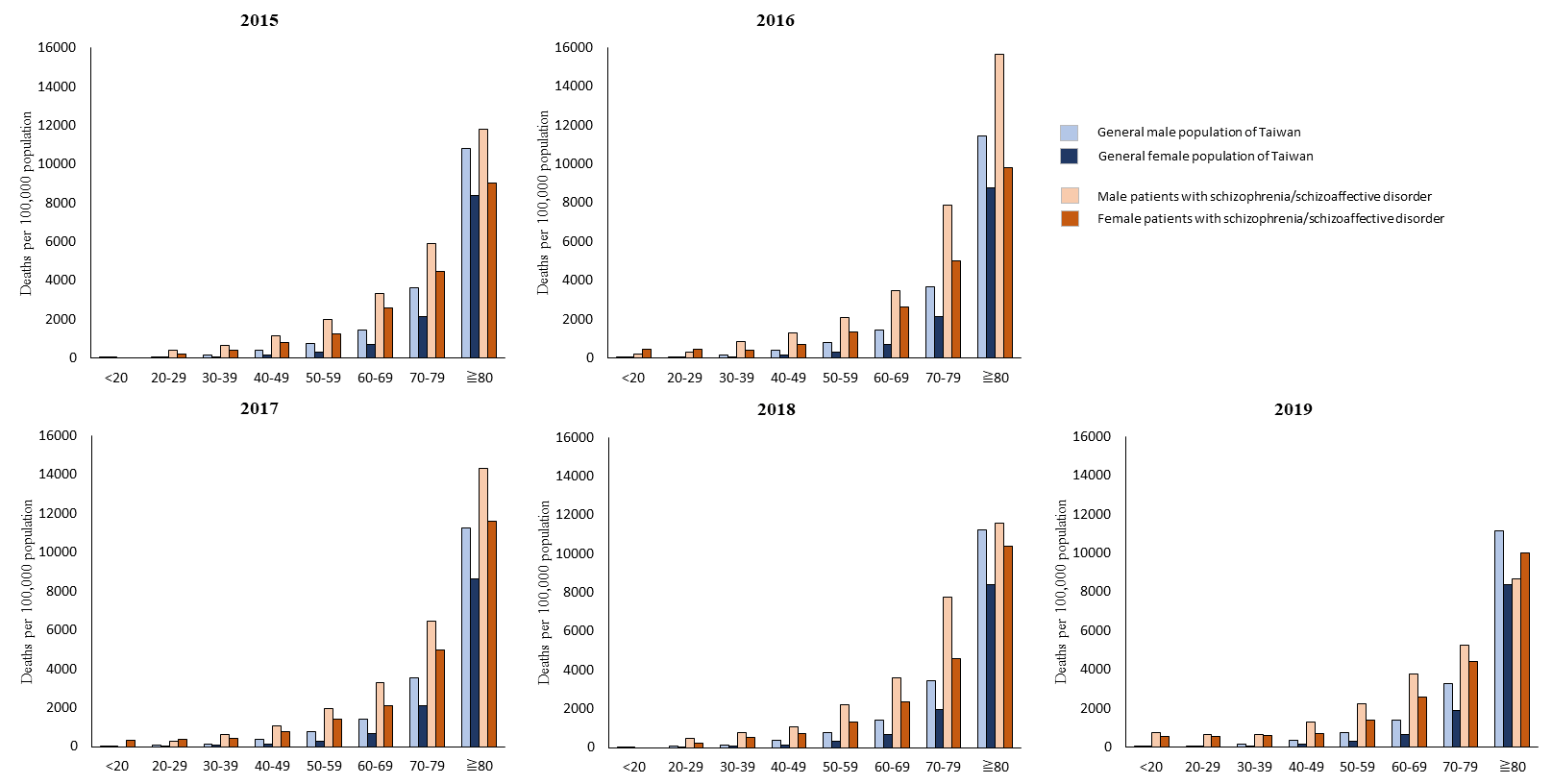
**
